# Assessment of infraorbital foramen location based on skeletal malocclusion, maxillary sinus pneumatization and infraorbital canal morphology using 3D CBCT scans

**DOI:** 10.1016/j.jobcr.2025.12.001

**Published:** 2025-12-06

**Authors:** Gülay Açar, Ahmet Safa Gökşan, Guldane Magat

**Affiliations:** a Department of Anatomy, Faculty of Medicine, Necmettin Erbakan University, Meram, 42090, Konya, Turkey; b Department of Anatomy, Faculty of Medicine, Aksaray University, Merkez, 68100, Aksaray, Turkey; c Department of Oral and Maxillofacial Radiology, Faculty of Dentistry, Necmettin Erbakan University, Meram, 42090, Konya, Turkey

**Keywords:** Safe infraorbital foramen location zone, 3D CBCT, Skeletal malocclusion, Maxillary sinus pneumatization, Infraorbital canal, Midface procedures

## Abstract

**Introduction:**

Understanding the infraorbital foramen (IOF) location is crucial in terms of regional anaesthesia as well as facial filler injection and radiofrequency neurotomy. We aimed to define a safe infraorbital foramen (IOF) location zone using three dimensional (3D) cone beam computed tomography (CBCT) scans and to evaluate the influence of skeletal malocclusion (SM), maxillary sinus pneumatization (MSP), and infraorbital canal (IOC) morphology on IOF position.

**Materials and methods:**

3D models of 300 (170 female, 130 male, range 18–50 years) CBCT scans were used to identify the SM, MSP, and IOC types, and to measure the distances from IOF to infraorbital margin, pyriform aperture, and zygion to determine IOF position. Using combined distances the IOF location zone was formed and divided into nine zones.

**Results:**

The IOF was predominantly located in the superocentral zones (Zones 1–6) in Class II patients, mostly associated with hypoplastic MS, Type I and IV IOCs. Conversely, in Class III patients mostly linked to hyperplastic MS, Type II and III IOCs, the likelihood of IOF was mainly observed in the inferocentral zones (Zones 4–9). Also, we observed a significant increase in the prevalence of Class I, hypoplastic MS, Type I and IV IOCs with age.

**Conclusion:**

Our results suggest that prediction of a safe IOF zone is possible based on SM, MSP, and IOC types in relation to gender and age within the studied population using 3D CBCT technology. This is essential for improved treatment planning and avoidance of iatrogenic injury during midface procedures.

## 1 Introduction

The infraorbital neurovascular bundle emerges from the infraorbital foramen (IOF), located below the infraorbital margin (IOM). Accurate identification of the IOF position is crucial for regional anaesthesia in midface, orbital and periocular procedures. To avoid iatrogenic injury, particularly in clinical settings, a predictive method for localising IOF is required.^1^, ^2^, ^3^, ^4^ The maxillary sinus (MS) is the largest and deepest of the paranasal sinuses, and its pneumatization is influenced by skeletal malocclusion (SM).^3^^,^^5^ Studies suggest that high infraorbital canal (IOC) protrusion/dehiscence, seen more often with higher degree of MS pneumatization (MSP), is linked to a longer septum and an elevated risk of traumatic injury, while reduced MSP may cause the IOF moving laterally and the orbital floor moving inferomedially.^6^^,^^7^ Furthermore, SM affects ventilation and mucus drainage in the MS, potentially causing facial asymmetries.^5^ Therefore, the SM, MSP, and IOC morphology are important factors that influence the IOF position, which is essential for the planning and efficacy of regional anaesthesia during midface surgery, facial filler injections and radiofrequency neurotomy of the trigeminal ganglion.^8^, ^9^, ^10^

Although studies have been done on IOF, IOC, and MS morphometry individually,^2^^,^^6^^,^^11^, ^12^, ^13^ not many show how IOF location can vary based on the SM, MSP, and IOC morphology. Cone beam computed tomography (CBCT) scans allow for an easy, detailed, three-dimensional (3D) assessment of the relationship between the IOF, IOC, MSP, and SM. We sought to identify a safe IOF location zone by measuring its distances to reference landmarks. We aimed to ascertain the impact of MSP and IOC morphology on IOF position by determining the prevalence of IOF in each zone and investigating any possible link between them, considering SM, gender, laterality, and age, using 3D CBCT scans.

## 2 Material and methods

This retrospective study was approved by the University's Ethical Committee (2024/5093) and was conducted using 300 CBCT images of randomly selected patients (170 female, 130 male, range 18–50 years). The sample size was estimated to be 250 using G∗Power version 3.1.9.7, with the 95 % confidence level (1 − α), 90 % test power (1 − β), and Cohen's f = 0.293 effect size and one-way analysis of variance (ANOVA) parameters.^3^

**Fig. 1:**
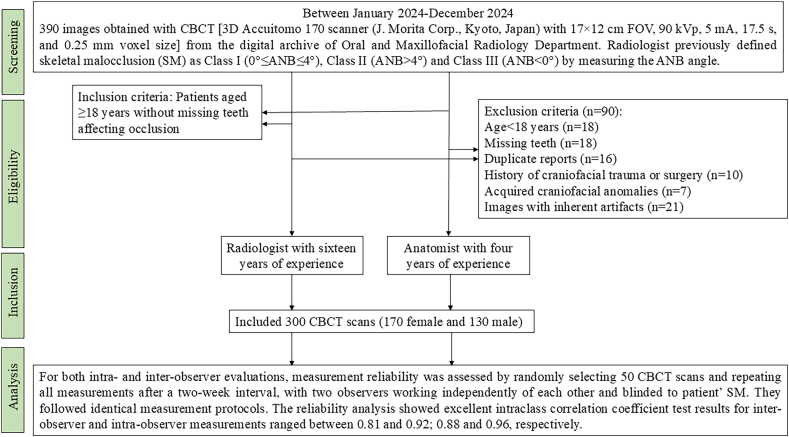
Study flowchart.

The patient recruitment and study design were illustrated in the form of a flowchart (Fig. 1). CBCT images were saved as DICOM files and transferred to 3D-Slicer software capable of reconstructing 3D volumetric models. To determine the IOF location, four reference landmarks including the IOF, pyriform aperture (PA), zygion (Z), and IOM were digitized in 3D CBCT images. We measured the distances from the IOF to these landmarks in coronal sections as follows;

IOF-PA: Horizontal distance between the medial border of the IOF and the lateral border of the PA,

IOF-IOM: Vertical distance between the superior border of the IOF and the lower margin of the orbit,

IOF-Z: Horizontal distance between the lateral border of the IOF and the most lateral point on the zygomatic arch.

**Fig. 2:**
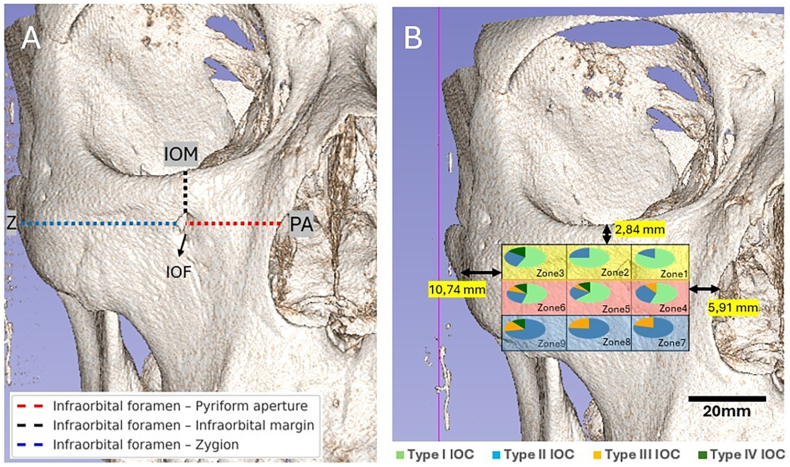
Identification of distance measurements (A) and safe infraorbital foramen location zones: yellow; superior zones, pink; central zones, blue; inferior zones (B). (For interpretation of the references to colour in this figure legend, the reader is referred to the Web version of this article.)

Excellent agreement (mean deviation ≤1 mm) was found for digitization of landmarks when two observers reviewed the annotations after completing each evaluation process, achieving full consensus on all interpretations. We used the combined values of these distances to form a rectangular safe IOF location zone, which was divided into nine equal zones as follows: Zone 1 (superomedial), Zone 2 (superocentral), Zone 3 (superolateral), Zone 4 (centromedial), Zone 5 (centrocentral), Zone 6 (centrolateral), Zone 7 (inferomedial), Zone 8 (inferocentral), and Zone 9 (inferolateral) (Fig. 2).

**Fig. 3:**
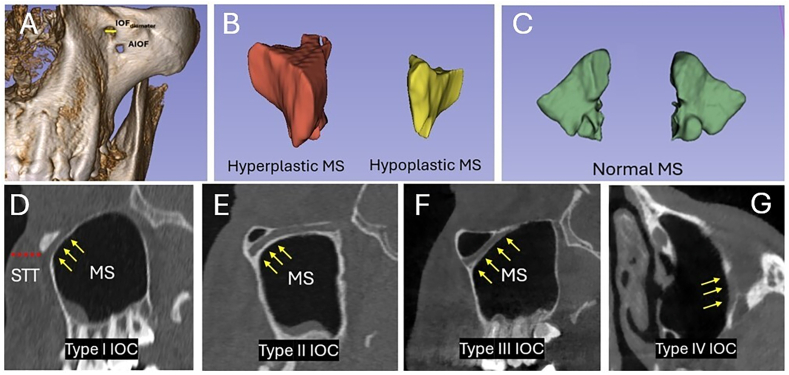
Identification of maxillary sinus and infraorbital canal types.

The IOF transverse diameter (IOF_diameter_) was measured and accessory IOF (AIOF) was also noted (Fig. 3A). 3D Slicer software automatically combined each section in which the boundaries of the MS were drawn to calculate MSV. According to MSV, MSP types were classified into hypoplastic MS (1–9.99 cm^3^), normal MS (10–19.99 cm^3^), and hyperplastic MS (20–32 cm^3^) (Fig. 3B and C). We identified four IOC types as follows; Type I (IOC within the MS roof), Type II (IOC under the MS roof), Type III (IOC protruded completely into the MS), and Type IV (show lateroantral course within the MS roof).^3^ On sagittal images soft tissue thickness (STT) was measured^14^ (Fig. 3D–G).

### 2.1 Statistical analysis

The data was analysed using Statistical Package for Social Sciences (SPSS, version 25.0). Intraclass correlation coefficient (ICC) was used to quantify intra- and inter-observer reliability. Gender and laterality differences were determined using the paired and unpaired t-tests. Morphometric data between the groups was analysed using ANOVA, while categorical data were compared using chi-square test. Multiple comparisons were performed with the Bonferroni post hoc test. p < 0.05 value was considered statistically significant.

## 3 Results

To better highlight the age-related changes, the sample was divided into four age groups: Group I (18–20 years, 123/300), Group II (21–25 years, 86/300), Group III (26–32 years, 48/300), and Group IV (33–50 years, 43/300). The mean MSV, IOF-PA, IOF-Z, IOF-IOM, STT, and IOF_diameter_ were measured as 14.85 ± 4.7 cm^3^, 14.89 ± 2.1 mm, 19.57 ± 3.1 mm, 7.40 ± 1.7 mm, 10.87 ± 2.3 mm, and 2.93 ± 0.6 mm, respectively. The detailed informations regarding the statistical analysis of the morphometric data based on gender, laterality, SM, MSP, and age groups were given in Table 1. The MSV and IOF-IOM were the highest in Class III. Hyperplastic MS and males had the largest value for all measurements except the STT. The MSV, IOF-PA, and IOF-IOM decreased, while the IOF_diameter_ and STT increased significantly with age, showing the most significant difference between Groups I and IV.

**Table 1:** Comparison of morphometric measurements according to gender, laterality, skeletal malocclusion, maxillary sinus pneumatization and age.

Morphometric data	MS volume(cm^3^) Mean ± SD	IOF-Pyriform aperture(mm) Mean ± SD	IOF-IOmargin(mm) Mean ± SD	IOF-Zygion(mm) Mean ± SD	IOF_diameter_(mm) Mean ± SD	Softtissue thickness(mm) Mean ± SD
**Female**	13.55 ± 4.3	13.23 ± 2.1	7.23 ± 1.6	18.95 ± 3.1	2.75 ± 0.5	10.81 ± 2.2
**Male**	16.53 ± 4.5	14.78 ± 2.2	7.54 ± 1.6	20.29 ± 3.3	3.16 ± 0.6	10.92 ± 2.4
**p value**	0.000∗∗∗	0.000∗∗∗	0,024∗	0.001∗∗	0.000∗∗∗	0.561
**95 %CI (lower-upper)**	−3.69─-2.26	−1.90─-1.18	−0.58─-0.04	−2.11─-0.55	−0.51─-0.31	−0.48─2.64

**Right**	14.91 ± 4.7	13.58 ± 2.2	7.0 ± 1.5	20.37 ± 3.3	2.95 ± 0.6	10.61 ± 1.9
**Left**	14.70 ± 4.6	14.01 ± 2.2	7.81 ± 1.6	20.95 ± 3.3	2.98 ± 0.6	10.88 ± 2.5
**p value**	0.067	0,013∗	0,924	0.105	0,020∗	0.089
**95 %CI (lower-upper)**	−0.01─0.42	−0.46─-0.05	−0.15─0.14	−0.05─0.53	−0.14─-0.01	−0.32─0.02

**Class I**	14.74 ± 4.7	14.07 ± 2.4	7.38 ± 1.9	20.40 ± 3.3	2.87 ± 0.6	11.10 ± 2.3
**Class II**	14.09 ± 4.1	13.58 ± 2.2	7.0 ± 1.5	20.37 ± 3.3	2.98 ± 0.6	10.61 ± 1.9
**Class III**	15.75 ± 5.0	14.01 ± 2.2	7.81 ± 1.6	20.95 ± 3.3	2.95 ± 0.6	10.88 ± 2.5
**p value**	0.001∗∗	0,180	0,002∗∗	0.634	0.238	0.104
**Bonferroni post hoc test**	ClassIII > ClassII	NS	ClassIII > ClassII	NS	NS	NS

**Hypoplastic MS(A)**	9.55 ± 2.6	13.31 ± 2.4	6.75 ± 1.5	19.44 ± 3.3	2.89 ± 0.6	11.52 ± 2.3
**Normal MS(B)**	15.0 ± 1.1	13.65 ± 2.1	7.44 ± 1.6	20.82 ± 3.6	2.89 ± 0.6	10.63 ± 2.3
**Hyperplastic MS(C)**	19.97 ± 2.4	14.73 ± 2.2	8.0 ± 1.8	21.43 ± 3.9	3.02 ± 0.7	10.48 ± 2.1
**p value**	0.000∗∗∗	0.000∗∗∗*-0.002∗∗*	0.000∗∗∗	0.023*∗*	0.103	0.001*∗∗*
**Bonferroni post hoc test**	C > B > A∗∗∗	C > A∗∗∗, C > B∗∗	C > B > A∗∗∗	C > A∗	NS	A > C∗∗, A > B∗∗

**Group I (18**–**20years)**	14.56 ± 4.9	15.34 ± 1.8	7.56 ± 1.6	19.87 ± 3.2	2.87 ± 0.6	10.43 ± 2.5
**Group II (21**–**25years)**	15.32 ± 4.3	14.75 ± 2.2	7.52 ± 1.8	20.96 ± 4.1	2.89 ± 0.7	10.84 ± 2.1
**Group III (26**–**32years)**	15.86 ± 4.1	14.62 ± 2.1	7.18 ± 1.5	21.44 ± 3.3	2.93 ± 0.6	10.86 ± 2.2
**Group IV (33**–**50years)**	13.60 ± 4.9	13.32 ± 2.5	6.94 ± 2.1	20.82 ± 2.9	3.11 ± 0.8	11.44 ± 2.7
**p value**	0.006∗∗*-0.031∗*	0.016∗	0.002∗∗-0.007∗∗	0.184	0.035*∗*	0.020*∗*
**Bonferroni post hoc test**	III > IV∗∗, II > IV∗	I > IV∗	I > IV∗∗, II > IV∗∗	NS	IV > I∗	IV > I∗

∗p < 0.05, ∗∗p < 0.01, ∗∗∗p < 0.001, NS not significant, CI confidence interval based on Student's t-test, Paired *t*-test, and ANOVA test results, MS maxillary sinus, IOF infraorbital foramen.

The most common SM was Class I (63 female, 41 male), followed by Class II (63 female, 34 male) and Class III (44 female, 55 male). Additionally, the most common was normal MS (216/600), followed by hypoplastic MS (192/600) and hyperplastic MS (192/604). The prevalence of hypoplastic and normal MS was higher in females, while hyperplastic MS was mostly observed in males (Table 2). Regarding IOC types, Type I was the most common (56.7 %), followed by Type II (29.5 %), Type III (9.8 %) and Type IV (4 %). There was no significant gender difference, whereas they were more prone to be bilateral, as were MSP types (p = 0.000). The prevalence of AIOF did not differ significantly with respect to gender, age, SM, MSP, and IOC types.

**Table 2:** Comparison of sample variants according to skeletal malocclusion and maxillary sinus pneumatization.

		Class I n(%)	Class II n(%)	Class III n(%)	Degrees of freedom	Cramer's V	p value	Chi-Square (χ^2^)
**Maxillary sinus pneumatization types**	Hypoplastic	72(34.6 %)	70(38.1 %)	50(25.3 %)				
	Normal	84(40.4 %)	66(32 %)	66(33.3 %)	4	0.104	0.011*∗*	12.956
	Hyperplastic	52(25 %)	58(29.9 %)	82(41.4 %)				
**Infraorbital canal types**	Type I	118(56.7 %)	130(67 %)	92(47.2 %)				
	Type II	60(28.8 %)	38(19.6 %)	80(39 %)				
	Type III	18(8.7 %)	14(7.2 %)	24(12.8 %)	6	0.134	0.002*∗*	21.477
	Type IV	12(5.8 %)	12(6.2 %)	2(1 %)				
**Accessory infraorbital foramen(IOF)**	Absent	128(61.5 %)	118(60.8 %)	132(66.7 %)				
	1	70(33.6 %)	64(33 %)	62(30.6 %)	4	0.051	0.800	3.071
	2	10(4.9 %)	12(6.2 %)	4(2.7 %)				
**Gender**	Female	63(60.6 %)	63(64.9 %)	44(44.4 %)				
	Male	41(39.4 %)	34(35.1 %)	55(55.6 %)	2	0.177	0.000∗	18.759

Maxillary sinus pneumatization types		HypoplasticMS n(%)	NormalMS n(%)	HyperplasticMS n(%)	Degrees of freedom	Cramer's V	p value	Chi-Square(χ^2^)
**Infraorbital canal types**	Type I	133(69.7 %)	123(56.9 %)	84(43.8 %)				
	Type II	31(15.3 %)	73(33.8 %)	74(38.5 %)				
	Type III	4(2.5 %)	20(9.3 %)	32(16.7 %)	6	0.268	0.000*∗*	86.447
	Type IV	24(12.5 %)	0(0 %)	2(1 %)				
**Accessory infraorbital foramen (IOF)**	Absent	122(63.5 %)	129(59.7 %)	127(66.1 %)				
	1	62(32.3 %)	77(35.7 %)	57(29.5 %)	4	0.072	0.407	6.150
	2	8(4.2 %)	10(4.6 %)	8(4.4 %)				
**Gender**	Female	67(69.8 %)	70(64.9 %)	33(34.4 %)				
	Male	29(30.2 %)	38(35.1 %)	63(65.6 %)	2	0.305	0.000∗	55.724

Chi-square test results, *∗* Statistically significant.

As shown in Table 2, the majority of cases of hypoplastic MS were observed in Class II, while hyperplastic MS was more prevalent in Class III samples. Meanwhile, Type I and IV IOCs were predominantly linked to hypoplastic MS, and Type II and III were mainly observed in cases of hyperplastic MS. In Table 3, there was a significant increase in the prevalence of Class I, hypoplastic MS, Type I and IV with age. The results revealed that being young and male was mostly associated with a higher likelihood of Class III and larger MSV and IOC protrusion.

**Table 3:** Comparison of sample variants according to age groups.

Age Groups		(18–20 years) n(%)	(21–25 years) n(%)	(26–32 years) n(%)	(33–50 years) n(%)	Degrees of freedom	Cramer's V	p value	Chi-Square (χ^2^)
**Skeletal** **Malocclusion**	Class I	39(31.7 %)	29(33.7 %)	15(31.3 %)	21(51.1 %)				
	Class II	43(35 %)	28(32.6 %)	13(27.1 %)	13(28.9 %)	6	0.106	0.036*∗*	13.451
	Class III	41(33.3 %)	29(33.7 %)	20(41.7 %)	9(20 %)				
**Maxillary sinus pneumatization types**	Hypoplastic	72(29.6 %)	50(28.5 %)	26(27.1 %)	44(51.2 %)	6	0.135	0.001*∗*	21.934
	Normal	100(41.1 %)	64(37.2 %)	32(32.7 %)	20(23.3 %)				
	Hyperplastic	72(29.3 %)	60(34.3 %)	38(40.2 %)	22(25.6 %)				
**Infraorbital canal types**	Type I	148(60.2 %)	94(54.7 %)	54(56.3 %)	44(51.2 %)				
	Type II	72(29.3 %)	52(30.2 %)	36(37.5 %)	18(20.9 %)				
	Type III	24(9.7 %)	22(12.8 %)	4(4.1 %)	6(7 %)	9	0.114	0.006*∗*	23.274
	Type IV	2(0.8 %)	4(2.3 %)	2(2.1 %)	18(20.9 %)				
**Accessory infraorbital foramen(IOF)**	Absent	154(62.6 %)	110(64 %)	64(66.7 %)	50(58.1 %)				
	1	76(30.9 %)	56(32.6 %)	30(31.2 %)	34(39.5 %)	6	0.075	0.351	9.998
	2	16(6.5 %)	6(3.4 %)	2(2.1 %)	2(2.4 %)				
**Gender**	Female	71(57.7 %)	54(62.8 %)	24(50 %)	21(48.8 %)				
	Male	52(42.3 %)	32(37.2 %)	24(50 %)	22(51.2 %)	3	0.105	0.085	6.623

**Infraorbital** **Foramen** **Location** **Zones**	Zone1	21(39 %)	18(33 %)	9(17 %)	6(11 %)	24	0.328	0.000*∗*	94.137
	Zone2	25(37 %)	22(33 %)	16(24 %)	4(6 %)				
	Zone3	20(29 %)	33(50 %)	14(21 %)	0(0 %)				
	Zone4	29(54 %)	14(26 %)	5(9 %)	6(11 %)				
	Zone5	67(67 %)	23(23 %)	6(6 %)	4(4 %)				
	Zone6	33(50 %)	21(32 %)	6(9 %)	6(9 %)				
	Zone7	25(66 %)	13(34 %)	0(0 %)	0(0 %)				
	Zone8	61(75 %)	21(25 %)	0(0 %)	0(0 %)				
	Zone9	59(83 %)	13(17 %)	0(0 %)	0(0 %)				

Chi-square test results, *∗* Statistically significant.

**Graph. 1:**
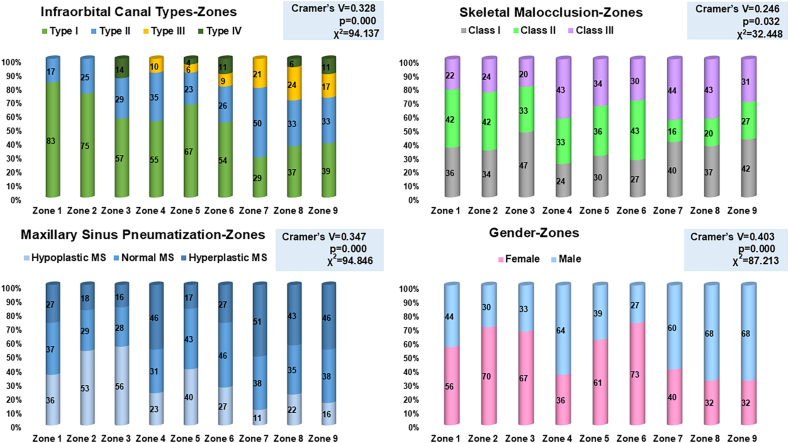
Showing study outcomes.

We measured the IOF-PA, IOF-IOM, and IOF-Z to be in a range of 5.5–20 mm, 2.5–11 mm, and 10.5–32 mm, respectively, in order to quantify a rectangular IOF location zone with dimensions of 10.71 mm × 39.32 mm positioning 5.91 mm, 10.74 mm and 2.84 mm from PA, Z and IOM (Fig. 2). Graph 1 gives the detailed distribution of the percentage of IOF location based on IOC types, SM, MSP and gender. The likelihood of the IOF location in superocentral zones (Zones 1–6) was higher in older females with Class II, hypoplastic MS, Type I and IV IOCs, while in younger males with Class III, hyperplastic MS, Type II and III IOCs, the IOF was mainly located in the inferocentral zones (Zones 4–9) (Table 3 and Graph 1).

## 4 Discussion

There is still no evidence as to whether the IOF position is guided by IOC morphology and MSP, which are often influenced by SM, gender, and ethnic variability. To our knowledge, no study has investigated this association in relation to gender, laterality, and age using 3D CBCT. Research in the field of literature has indicated that variations in the morphology of paranasal sinuses and position of craniofacial structures can arise due to alterations in the developmental anatomy of the craniofacial region. These alterations can be attributed to either airway obstruction or changes in the direction of craniofacial growth in different SM.^7^^,^^15^ Our results revealed a significant influence of SM and gender on MSV, in which Class III males mostly had higher MSV than females in other Classes, aligning with Lessa et al.^15^ but contrasting with Shrestha et al.^7^ who stated that Class II had higher MSV. Thus, it appears that the forward placement of the larger mandible in Class III males may result in a larger maxilla with higher MSV value. Previous studies indicated that hypoplastic MS is linked to an increased risk of Type I and IV IOCs, while the probability of higher MSV is decreased by ageing and being female.^3^, ^4^, ^5^, ^6^ Current studies reported that adolescent males with higher MSV are more likely to have Type III IOC, and older females are more likely to have hypoplastic MS, with a higher prevalence of Type I IOC.^3^^,^^4^ The presence of Class II alongside Types I and IV IOCs may be attributed to hypoplastic MS, which hinders IOC protrusion. Conversely, hyperplastic MS was mostly associated with Types II and III IOCs, which protrude to a greater degree depending on higher MSV. We also noted that the STT was significantly higher in hypoplastic MS and old group, which may be due to resorption and recession of the maxilla. Although the exact mechanism of AIOF formation is not clear, the prevalence of it was found to be 37 % (222/600), similar to previous reports ranging from 16.9 % to 47.6 %.^11^, ^12^, ^13^, ^14^^,^^17^

We suspected there may be huge differences in the localization of the IOF depending on SM, MSP, and IOC types in relation to gender and age. A 3D model of the IOF zone was successfully constructed with safe margin limits of 14.30mmx10.60 mm, located 3.50 mm below the IOM, 7.10 mm medially from the PA, and 11.60 mm laterally from Z, aligning with Aseem et al.‘s study.^16^ Furthermore, we divided this zone into nine subzones and identify each zone's IOF frequency in relation to SM, MSP, IOC, gender, and age.

**Graph. 2:**
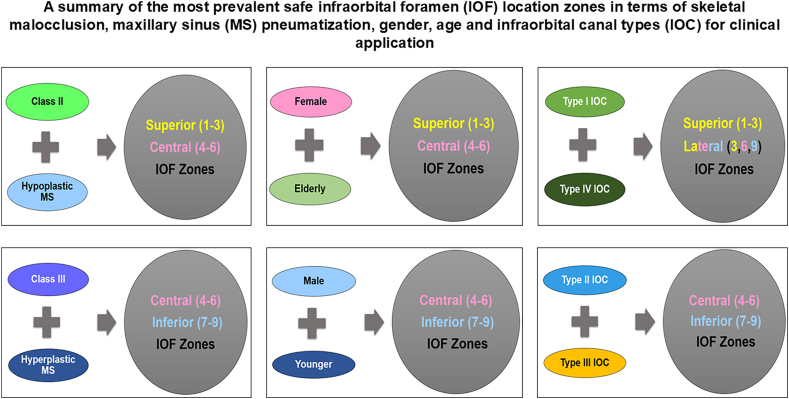
Summary of study outcomes.

We found that the IOF location in inferocentral zones (Zones 4–9) was predominantly observed in Type II and III IOCs, which were mostly associated with hyperplastic MS and Class III malocclusion, particularly in younger males. In contrast, Type I and IV IOCs, along with their superocentrally (Zones 1–6) and laterally (Zones 3,6,9) positioned IOF, may be linked to hypoplastic MS and Class II malocclusion, primarily in older females (Graph 2).

Our findings can be used to accurately specify a safe IOF zone. This is important for helping clinicians to achieve patient satisfaction through more effective anaesthesia, as well as for preventing iatrogenic injury during orbital and midface procedures. Clinicians should anticipate an inferocentrally positioned IOF and adapt injection approaches accordingly for patients with Class III malocclusion and hyperplastic MS. For female patients exhibiting Type I and IV IOCs in conjunction with hypoplastic MS, superocentrally and laterally positioned IOF should be considered. Furthermore, elderly patients, especially females, are more likely to develop hypoplastic MS, causing superolateral displacement of the IOF and inferomedial positioning of the orbital floor.

To our knowledge, this is among the first studies to simultaneously evaluate SM, MSP, and IOC morphology in relation to IOF location using 3D CBCT. Therefore, we could not find any studies to compare our results with. While our study corroborates many previous anatomical discoveries, it takes a novel approach, integrating SM, MSP, IOC morphology, gender, and age into a single framework, offering a considerably more complex portrayal of the impact of these factors on IOF location, extending beyond the scope of earlier researches. Furthermore, several new anatomical insights concerning the classification and prevalence of MSP and IOC types have been revealed, and confidence in the results has been increased by a relatively large statistically powered sample. The use of 3D CBCT allowed for better visualization, improving reproducible and precise measurements and variant evaluation in living subjects, which can be applied clinically. This study is not just an anatomical description, but a clinically applicable baseline framework. The presence of demographic factors, including gender and age, provides nuanced insights for the planning of personalized surgeries.

This retrospective study has some limitations. They include a lack of clinical or surgical data, a single-centre design of healthy patients, and restriction to a specific racial group, limiting its generalisability. Further multicenter, prospective cohort studies comparing unhealthy and control groups across a wide age range are needed to validate this clinically applicable framework in different ethnic groups.

## 5 Conclusion

Our results suggest that prediction of a safe IOF zone is possible based on SM, MSP, and IOC types in relation to gender and age within the studied population using 3D CBCT technology. A high level of doubt regarding inferocentrally positioned IOF might be sustained for Class III younger males who mainly display Type II and III IOCs in hyperplastic MS, while superocentral IOF location may be presumed for Type I and IV IOCs linked mostly with hypoplastic MS in Class II older females. In clinical practice, this may facilitate predicting the location of the neurovascular bundle, enabling effective regional anaesthesia to be administered and avoiding iatrogenic injury during midface procedures. Therefore, when planning regional anaesthesia during midface surgery, facial filler injections and radiofrequency neurotomy, SM, MSP, IOC morphology, gender and age should be considered.

## Patient_spl_s guardian

The need for informed consent was waived by the Research Ethics Committee of Necmettin Erbakan University Faculty of Medicine due to the study's use of retrospective and anonymized patient information obtained from electronic records.

## Author contributions

Conceptualization G.A., G.M., data collection: A.S.G., Statistical analysis: G.A., data interpretation: A.S.G., G.M., writing the first draft: A.S.G., G.A., Manuscript editing and reviewing: All authors.

## Ethical clearance

This retrospective study was approved by the Drug and Non-Medical Device Research Ethics Committee of Necmettin Erbakan University Faculty of Medicine (approval number 2024/5093). This study was carried out in compliance with the principles of the Helsinki Declaration, relevant Turkish legislation, and General Data Protection Law regulations on medical research.

## Funding

No funding

## Declaration of competing interest

The authors declare that they have no known competing financial interests or personal relationships that could have appeared to influence the work reported in this paper.
